# Two or more enteropathogens are associated with diarrhoea in Mexican children

**DOI:** 10.1186/1476-0711-6-17

**Published:** 2007-12-28

**Authors:** Gloria Luz Paniagua, Eric Monroy, Octavio García-González, Javier Alonso, Erasmo Negrete, Sergio Vaca

**Affiliations:** 1 Facultad de Estudios Superiores Iztacala, Universidad Nacional Autónoma de México, Avenida de Los Barrios. 1, Los Reyes Iztacala, Tlalnepantla, 54090, Estado de Mexico, Mexico

## Abstract

**Background:**

Diarrhoeal diseases constitute a major public health problem, particularly in the developing world, where the rate of mortality and morbidity is very high. The purpose of this study was to conduct a 2 years and 3 months study in order to determine the prevalence of five enteropathogen diarrheogenic agents in Mexico City.

**Methods:**

Faecal samples were obtained from 300 Mexican children diagnosed as positive for diarrhoea, aged > 2 to < 12 years old, and from 80 children matched for age but with no symptoms of the disease (control group). Two multiplex PCR were used to detect *Escherichia coli*, *Salmonella *spp., and *Shigella *spp. In addition, the two protozoan parasites *Entamoeba histolytica/Entamoeba dispar *and *Giardia intestinalis *were detected by conventional methods.

**Results:**

All diarrhoeal samples were positive for one or more enteropathogens. The most common enteropathogens in diarrhoeal samples were *E. histolytica/E. dispar *(70.3%), *Salmonella *(*ohio *28.3%; *typhimurium *16.3%; *infantis *8%; *anatum *0.6%; Newport 0.3%), *G. intestinalis *(33%), *E. coli *(ETEC 13.3%; EPEC 9.3%; VTEC 8.6%; EIEC 1%) and *Shigella *spp. (*flexneri *1.6%, *sonnei *1%). Infections by two (24%) three (16%) and four (12%) pathogens were observed.

**Conclusion:**

This study revealed that 52% of the patients were infected by more than one enteropathogen, notably *E. histolitica*/*E. dispar *and *Salmonella ohio*. These results are useful for clinicians to improve the empiric treatment used in such cases.

## Background

Diarrhoeal diseases constitute a major public health problem, particularly in the developing world, where the rate of mortality and morbidity is very high [1]. The World Health Organization (WHO) has estimated that 1.5 billion episodes of diarrhoea occur every year in developing countries, resulting in 3 million deaths [2]. In Mexico, a governmental study conducted in the year 2003 reported 4556 cases caused by intestinal infectious [3]. The etiological agents of diarrhoea described in epidemiological studies are transmitted as waterborne and foodborne.

Some foodborne pathogens have been recently considered as emerging diseases [4], despite the fact they have been known since a long time ago. For example, outbreaks of salmonellosis have been described for many decades, and yet their incidence have increased over the last 25 years. Diarrhoeal infections can be caused by many etiological agents, but mainly by enterobacteria such as *Escherichia coli, Salmonella *spp., *Shigella *spp., *Campylobacter jejuni *and *Vibrio cholerae*; as well as parasites such as *Entamoeba histolytica *and *Giardia intestinalis*, and some rotaviruses are also important agents [5].

*Escherichia coli *is considered as the etiological agent for many diseases including some affecting the urinary tract and intestine. The classification of diarrhoegenic *E. coli *strains is based on their virulence properties, and comprises six groups: Enterotoxigenic *E. coli *(ETEC), Enteropathogenic *E. coli *(EPEC), Enteroinvasive *E. coli *(EIEC), Enterohaemorragic *E. coli *(EHEC), Enteroaggregative *E. coli *(EAggEC) and Diffuse Adhering *E. coli *(DAEC) [6]. *Salmonella *spp. is a facultative, gram negative, flagellated member of the Enterobacteriaceae family. The most extensive accepted classification of *Salmonella *strains is based on the diversity of two differentially expressed H flagellar antigens: flagellin phase I and phase II antigens (codified by *fli*C and *flj*B genes), and the O antigens of the bacterial lipopolysaccharide, both determined by serotyping [7]. Until now, 2501 serotypes have been described [8]; which turns *Salmonella *classification into a complex and laborious process in the clinical laboratory; therefore, several PCR based methods have recently been developed, and were reported to be a simple, highly sensitive, fast and reliable alternative when compared to traditional clinical laboratory methods [9,10].

*Shigella *is a Gram negative, non-motile, rod-shaped bacteria, closely related to *E. coli *and *Salmonella*, and it is the etiological agent of human shigellosis and dysentery, which is characterized by severe diarrhoea with the presence of blood in the faeces. Classification of *Shigella *is based on serotyping, and comprise the following groups and serotypes: Serogroup A (*S. dysenteriae*) 12 serotypes, serogroup B (*S. flexneri*) 6 serotypes, serogroup C (*S. boydii*) 23 serotypes and serogroup D (*S. sonnei*) with only one serotype [11].

Diarrhoea can also be caused by protozoa such as *Entamoeba histolytica *and *Giardia intestinalis*, these zoonotic parasites are frequently transmitted by consumption of water contaminated with infective cysts [12].

The purpose of this study was to determine the prevalence of five enteropathogen diarrheogenic agents namely *E. coli*, *Salmonella *spp., *Shigella *spp., *E. histolytica/E. dispar *and *Giardia intestinalis *in Mexico City. Stool specimens obtained from children patients from several communities in Mexico City were examined; the association patterns between different pathogens and its correlation with occurrence of diarrhoea were also described.

## Materials and methods

### Stool samples

A total of 300 stool samples were obtained from children patients with diarrhoea from different hospitals in Mexico City (patient group); also, 80 samples were obtained from children attending schools in the surrounding area, who did not had diarrhoea in the previous 45 days (control group). All subjects aged > 2 to < 12 years, and samples were collected from September 2004 through December 2006.

The selection criteria for inclusion of children patients with diarrhoea was having at least 3 or more soft, semisolid or liquid bloody faeces within 24 hours. Also, selection was made on the basis of a questionnaire filled up by all subjects with assistance of a relative over 18 years old, providing information regarding other gastrointestinal disorders, non-related diseases, travelling, frequency of diarrhoeal episodes, previous and current antibiotic treatment; as well as general data such as age, gender and place of residence. The control and patient groups were matched for age and sex.

### Bacteriology

Stool samples were streaked on the surface of MacConkey agar (DIBICO, Mexico) for obtaining *E. coli *isolates and on sodium deoxycholate agar for the selection of *Shigell*a and *Salmonella*, and were incubated overnight at 37°C. All samples were tested for *Shigella *by using colony morphology, biochemical properties, and agglutination with specific antisera (Serobac, BioRad).

Multiplex PCR assays for the detection of *E. coli *and *Shigella *spp. were performed following a previously reported method [13], which selectively amplifies specific regions of several virulence genes: ETEC (*eltB *322 bp, and *estA *147 bp) VTEC (*eaeA *376 bp, *vt1 *130 bp, and *vt2 *298 bp), EPEC (*eaeA *376 bp, and *bfpA *367 bp) EIEC (*ial *320 bp), *E. coli *O157:H7 (*fliC *H7 625 bp, and O157, 500 bp) present in diarrhoegenic *E. coli*; however, although this method is also capable of detecting *Shigella *spp., it can not distinguish it from EIEC, since the amplification target used by this method is a region of the invasion-associated locus (*ial*), common to both species. Therefore, the presence of *Shigella *spp. was also confirmed by using specific antisera.

In order to molecularly serotype *Salmonella *spp., we used two previously described methods [9,10], which makes use of the ability of *Salmonella *spp. to differentially express *fli*C gene (phase I) and *flj*B (phase II) flagellar H antigens, allowing the identification of the corresponding DNA variable internal regions (H:i, H:r, H:I, v, H:e, h, H:z_10_, H:b, H:d, for phase I; and H:1,2, H:1,5, H:1,6, H:1,7, H:I, w, H:e, n, x and H:e, n, z_15 _for phase II).

Positive controls containing template DNA of the following reference strains were used in every amplification round: ETEC ATCC 35401; EPEC ATCC 43887, EHEC ATCC 43890, EHEC ATCC 43889, EIEC ATCC 43893, *E. coli *ATCC 11775 (negative control without virulence genes), *Salmonella typhimurium, Salmonella paratyphi *B and *Salmonella infantis*. All primers used were obtained from Sigma Genosys (Sigma). Gel electrophoresis was photodocumented using a Gel Logic 100 Imaging system (KODAK). Molecular sizing of the amplicons was performed using KODAK Molecular Imaging Software.

### Parasitology

Determination of *E. histolytica/E. dispar *and *G.intestinalis *was done using Faust method. Protozoa were concentrated by centrifugal-floatation (500 × g 2 min) using zinc sulphate as the diluent (specific gravity 1.19) and observed with a light microscope at 40× [14].

### Statistical methods

Differences between isolation rates among patient and control groups were evaluated by the χ^2 ^test.

## Results

### Stool samples

Sex distribution was similar in both groups: 55% of population were females and 45% were males. Sex distribution did not show to play an important role regarding enteropathogen prevalence. Regarding the patient group, 58% presented abdominal pain, vomit and fever (> 39°C); and 20% required oral rehydration. Ten percent of the stool samples obtained from this group presented blood.

### Identification of enteropathogens

In the patient group, the predominant enteropathogen was *E. histolytica/E. dispar *(70.3%), followed by *G. intestinalis *(33%), *S*. *ohio *(28.3%), *S. typhimurium *(16.3%) and ETEC (13.3%); regarding the control group, *E. histolytica/E. dispar *(43.7%) was also the most commonly founded enteropathogen (Table 1), nevertheless, the prevalence of all detected enteropathogens was significantly different (P < 0.001).

**Table 1:** Prevalence of enteropathogens among the studied populations.

**Enteropathogen**	**Patient group (%) n = 300**	**Control group (%) n = 80**
*Entamoeba histolytica/Entamoeba dispar*	211 (70.3)	35 (43.7)
*Giardia intestinalis*	99 (33.0)	16 (20.0)
*Salmonella ohio*	85 (28.3)	2 (2.5)
*Salmonella typhimurium*	49 (16.3)	1 (1.2)
ETEC	40 (13.3)	2 (2.5)
EPEC	28 (9.3)	1 (1.2)
*Salmonella infantis*	24 (8.0)	0
VTEC	26 (8.6)	1(1.2)
*Shigella flexneri*	5 (1.6)	0
*Shigella sonnei*	3 (1.0)	0
EIEC	3 (1.0)	0
*Salmonella anatum*	2 (0.6)	0
*Salmonella *Newport	1 (0.3)	0

ETEC, enterotoxigenic *E. coli; *EPEC, enteropathogenic *E. coli; *VTEC, verotoxigenic *E. coli; *EIEC, enteroinvasive *E. coli*.

The predominant pattern was comprised by a single enteropathogen infection (48%), followed by co infection involving two pathogens (24%), and being the less common co infection by four pathogens (12%) (Table 2).

**Table 2:** Association patterns of enteropathogens in patient group stool samples.

**Four Pathogen infection**	n*
*E. histolytica*/*E. dispar *+ *G. intestinalis *+ *S. typhimurium *+ ETEC LT	2
*E. histolytica*/*E. dispar *+ *G. intestinalis *+ *S*. *ohio *+ EPEC	2
*E. histolytica*/*E. dispar *+ *G. intestinalis *+ *S*. *ohio *+ ETEC LT	2
*E. histolytica*/*E. dispar *+ *G. intestinalis *+ *S*. *ohio *+ *S*. *typhimurium*	2
*G. intestinalis *+ *S*. *ohio *+ ETEC LT-ST/*S. infantis*	2
*E. histolytica*/*E. dispar *+ *S*. *ohio *+EPEC + *S. infantis*	2
*E. histolytica*/*E. dispar *+ *G. intestinalis *+ *S*. *ohio *+ VTEC	2
Subjects with different associations	22

**Three Pathogen Infection**	

*E. histolytica*/*E. dispar *+ *S. infantis *+ VETEC	2
*E. histolytica*+ *S. typhimurium *+ VETEC	2
*E. histolytica*+ *G. intestinalis *+ EPEC	2
*E. histolytica*+ *S*. *ohio *+ EPEC	2
*E. histolytica*+ *S*. *ohio *+ VETEC	2
*E. histolytica*+ *S*. *ohio *+ *S*. *typhimurium*	2
*G. intestinalis*+ *S*. *ohio *+ VTEC	2
*E. histolytica*+ *S*. *ohio *+ EPEC	3
*E. histolytica*+ *G. intestinalis*+ S. *typhimurium*	7
*E. histolytica*+*G. intestinalis *+ *S*. *ohio*	7
*E. histolytica*+ *G. intestinalis*+ *S*. *ohio*	7
Subjects with different associations	10

**Two Pathogen Infection**	

*E. histolytica*/*E. dispar *+ EPEC	2
*G. intestinalis *+ EPEC	2
*S. ohio *+ *S*. *typhimurium*	2
*E. histolytica*/*E. dispar *+ *S. anatum*	2
*G. intestinalis *+ *S*. *typhimurium*	2
*E. histolytica*/*E. dispar *+ VTEC	3
*E. histolytica*/*E. dispar *+ *S. infantis*	3
*G. intestinalis *+ ETEC LT	3
*E. histolytica*/*E. dispar *+ *S*. *typhimurium*	6
*E. histolytica*/*E. dispar *+ *S*. *ohio*	14
*E. histolytica*/*E. dispar *+ *G. intestinalis*	23
Subjects with different associations	10

*n: number of subjects positive for indicated enteropathogens; abbreviations are the same as indicated in Table 1.

In the control group, 27.5% stool samples were free of any of the bacterial enteropathogens analyzed, 62.5% were positive for one and 10% were positive for two enteropathogens (*E. histolytica/E. dispar *and *G. intestinalis*).

### Multiplex PCR and detection of Enterobacteriaceae

Only 195 *E. coli *strains were detected in the stool samples (160/300 from patient group and 35/80 from control group); 46.6 % (140/300) of cases and 56.2% (45/80) of controls stools contain no *E. coli*. Analysis by multiplex PCR shows that only 101 out of 195 *E. coli *were positive (97/160, 60.6%) from patients and 4/35 (11.4%) from control group. Eight isolates from patients were further identified as *Shigella *(Table 1). The prevalence of diarrhoegenic *E. coli *in both groups was significantly different (P < 0.001). The frequencies of the diarrhoegenic *E. coli *positive for one of the targeted genes are shown in Table 3.

**Table 3:** Diarrhoegenic *E. coli *identified in stool samples.

**Virulence genes amplified**	**Patient group (%) n = 300**	**Control group (%) n = 80**
ETEC		
*estA*	10 (3.3)	1 (1.2)
*eltB*	20 (6.6)	1 (1.2)
*estA + eltB*	10 (3.3)	0

EPEC		
*eaeA*	23 (7.6)	1 (1.2)
*eaeA + bfpA*	5 (1.7)	0

VTEC		
*vt1 + vt2 + eaeA*	17 (5.6)	1 (1.2)
*vt2 + eaeA*	5 (1.6)	0
*vt1 + eaeA*	4 (1.3)	0

EIEC		
*ial*	3 (1.0)	0

Total	97 (32)	4(5)

χ^2 ^represents statistical difference between both groups studied (P < 0.001). Abbreviations are the same as indicated in Table 1.

Regarding *Salmonella *spp., the most common species in the patient group was *S*. *ohio *(28.3%), followed by *S*. *typhimurium *(16.3%) and *S. infantis *(8.0%); compared with *S. ohio *(2.5%) and *S*. *typhimurium *(1.2%) in the control group (Table 1).

## Discussion

The current study used a variety of diagnostic methods which helped estimate 100% prevalence of the enteropathogens in stool samples from children presenting diarrhoea symptoms. In addition, a high rate for multiple infections 156/300 (52%) was observed. However, not only the patient group had enteropathogens, also 50/80 (62.5%) members of the control group were positive for either one or two parasites (*E. histolytica/E. dispar *and *Giardia intestinalis*). Although only 4 (5%) of the control group were positive for diarrhoegenic *E. coli *(Table 3), they did not have any symptoms by the time this study was conducted. One of the strains detected in the controls was *E. coli *O157:H7, which has been recently described as an emerging pathogen worldwide [6,15]. The high rate of enteropathogens detected in this study among both groups, reflects the importance of monitoring on a daily basis the most vulnerable population, such as low economic level children groups.

*E. histolytica/E. dispar *were the most frequently found enteropathogen in both, the patient (70.3%) and the control group (43.7%) (Table 1). Globally, amoebiasis is wide spread in approximately 20% of the world's population; 10% of those individuals get sick, and 0.1- 0.25% of them die. *E. histolytica *constitutes the third cause of death for parasitic diseases [16]. On the other hand, *G. intestinalis *a protozoa which causes symptomatic infections mainly in children under 12 years old was observed in 33% of the patient group, and 20% for the control group (Table 1). These results are in agreement with a previous study that reported *G. intestinalis *in 29.9% of a population group in Mexico City [17]. An interesting observation is that although 63.7% of the members of the control group were positive for *E. histolytica/E. dispar *and/or *G. intestinalis *(Table 1), none of them presented symptoms of disease.

**Figure 1:**
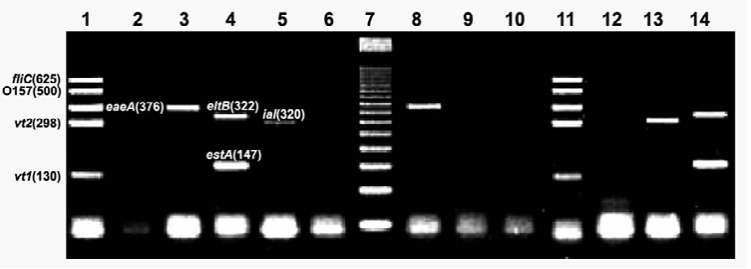
2.5% Agarose gel electrophoresis of *E. coli *amplicons obtained from diarrhoeal samples (run conditions 120 V, 94 mA, 45 min.). Lane 1: VTEC ATCC43889 and ATCC43890 strains (positive control). Lane 2: Negative control without template DNA. Lane 3: EPEC ATCC43887 strain (positive control). Lane 4: ETEC ATCC 35401 (positive control); Lane 5: EIEC ATCC43893 (positive control). Lane 6: *E. coli *ATCC11775 (avirulent strain, negative control). Lane 7: MWM 50-bp ladder. Lane 8: EPEC (patient group sample). Lane 9: negative sample (control group). Lane 10; negative sample (control group). Lane 11; VTEC (patient group sample). Lane 12; negative sample (patient group). Lane 13; EIEC (patient group sample). Lane 14; ETEC (patient group sample).

We used three multiplex PCR methods that accurately detected specific genetic virulence markers of *Salmonella *spp., *Shigella *spp., and *E. coli *groups ETEC (*eltB *and *estA *gene fragments), EPEC (*eaeA *and *bfpA *gene fragments), VTEC (*eaeA *and *vt1*or *vt2 *genes), EIEC/*Shigella *(*ial *region), and *E. coli *O157:H7 (*fliC *H7 flagellar antigen and a characteristic O157:H7 DNA fragment previously described) [18]. EIEC and *Shigella *are closely related regarding some of their virulence factors and phenotypic properties [19,20], the multiplex PCR method employed in this study can not be used alone for a definitive discrimination between these two enteropathogens since it recognizes the *ial *locus, which can be found in both, EIEC and *Shigella *spp. Therefore, *ial *positive amplification isolates were considered as positive for EIEC if they showed no agglutination when challenged against specific antiserum for *Shigella *spp. Also, because *elt*B and *ial *amplicons are very close regarding their molecular size (322 and 320 bp respectively) (Fig. 1), two separate PCR reactions were done, each containing specific primers against *elt*B or *ial *in order to avoid the overlapping of amplification products, that might lead to false positives.

Diarrhoegenic *E. coli *was present in 32% of the patient group and 5% of the control group (Table 3); these data are consistent with a previous study conducted in Hanoi, Vietnam, where diarrhoegenic *E. coli *was detected by multiplex PCR, in 22.5 % of the patient group and 12% for the control group [21]. The presence of diarrhoegenic *E. coli *groups has been reported world wide and recognized as one of the major causes of deceases involving enteropathogens in children [22-26]. In Mexico and other developing countries in the world, ETEC is the most prevalent diarrhoegenic *E. coli *group [27,28]. It is considered as an important pathogen in children, especially during the first six months, where the isolation rate ranges from 10 to 30% [29]. In this study, the presence of ETEC in the patient group (13.3%) was the highest compared to the other *E. coli *groups detected (Table 1). However, this rate is lower than what has been reported in some parts of the world, which reaches up to 20.7% [30,31]. It seems that, regardless of the location, the presence of *eltB *gene (encoding thermo labile toxin) is a definitive advantage for ETEC, since in our study, the virulence gene distribution rate for *eltB *was high (6.6%). The same observation was reported by another studies conducted in Sweden [13] and Vietnam [21], where *eltB *was the most commonly found ETEC virulence gene in the studied groups. The second most commonly found diarrhoegenic *E. coli *strain was EPEC (9.3%), which has been reported responsible for a high rate of mortality and morbidity among children, especially in developing countries where poor sanitary conditions prevail [6]. For example, in countries such as Mexico [32,33] and Brazil [34] up to 40% of diarrhoeal episodes in children are due to EPEC. Interestingly, regarding virulence gene homogeneity distribution, it seems that the situation for EPEC is not the same as for ETEC. A higher percentage of *eae*A+/*bfp*A- isolates (atypical strains) was found compared to *eaeA *+*bfpA+ *(typical strains) (Table 3), which differs from others studies conducted in Vietnam, where most of the isolates were *eaeA *+*bfpA+ *[21].

VTEC *E. coli*, an enteropathogen distributed worldwide, that has been more extensively studied in USA [35] and Europe [36] was also detected in this study. One strain of VTEC was detected in the controls whereas in the patient group it had a prevalence rate of 8.6% (26/300) (Table 1), of which 17/26 (65.4 %) possessed *vt1*, *vt2 *and *eaeA; *19.2% (5/26) possessed *vt2 *and *eaeA *and 15.3% (4/26) *vt1 *and *eaeA; *being *vt1 *the most abundant genotype within this group (Table 3). These results differ from those reported by Svenungsson *et al*., [13] where *vt1 *was in fact the less commonly found genotype in their samples.

*E. coli *O157:H7 is an emergent pathogen causing the haemolytic – uremic syndrome, and is considered as an important foodborne source of intestinal infection causing more than 73,000 diarrhoeal episodes in the United States every year [15,37]. We found one *E. coli *O157:H7 in the control group, however, no symptoms of diarrhoea were reported by the time this study was conducted. There is no explanation for this, but it might be possible that the infection was in an early stage when the stool sample was collected, or that the O157:H7 was a non-producing toxin strain. EIEC was the least detected in the patient group (1%) (Table 1), which is in agreement with a previous study conducted in a surrounding area of Mexico City where prevalence was 0.85% [28]. It has been estimated that EIEC in developing countries including Latin American is rare [5,38]. The prevalence rate for *Shigella sonnei *(1.6%) and *Shigella flexneri *(1%) in the current study was very low (Table 1) when compared other studies [13,21]. However, in other parts of the world, several *Shigella *outbreaks have been described, and the main feature is their ability to spread in the population, due to its very low infection doses (~10 bacterial cells per host) [19,20]; therefore, a low prevalence of cases of *Shigella *should not be underestimated.

**Figure 2:**
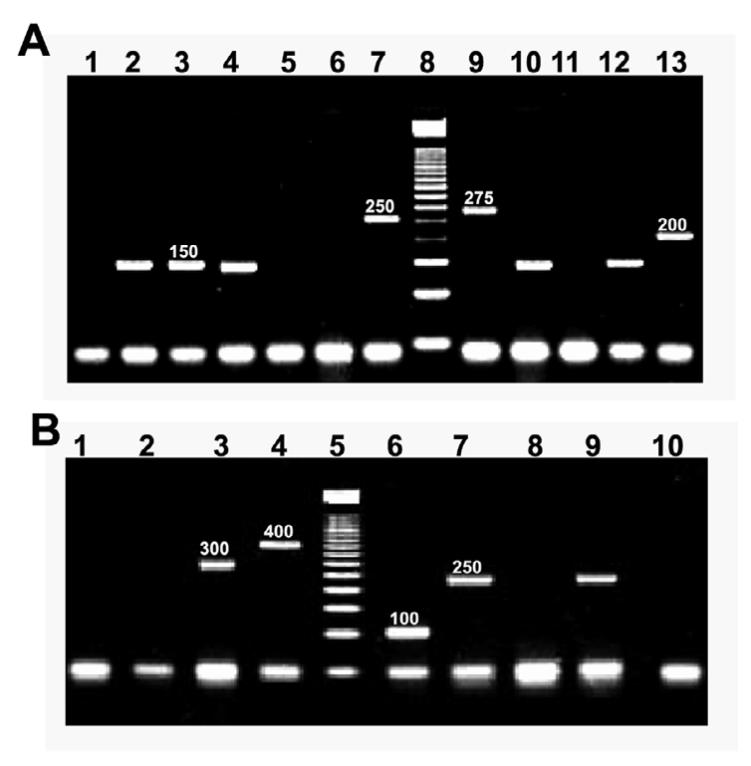
**(A) **2.5% Agarose gel electrophoresis of *Salmonella *phase I flagellar amplicons obtained from diarrhoeal samples. Lane 1: Negative control without template DNA. Lanes 2, 3, 4 10 and 12; *S. ohio *(150 bp) (patient group). Lanes 5, 6 and 11; negative samples (patient group). Lane 7 *S. typhimurium *(patient group). Lane 8; MWM 50 bp ladder. Lane 9; *S. infantis *(patient group); Lane 11; Negative sample (patient group); Lane 13; *Salmonella anatum *(patient group). **(B) **2.5% agarose gel electrophoresis of *Salmonella *phase II flagellar amplicons obtained from diarrhoeal samples. Lanes 1, 8 and 10, negative samples (patient group); Lane 2, Negative control without template DNA; Lane 3, *Salmonella anatum *(patient group); Lane 4, *S. typhimurium *(patient group); Lane 5, MWM 50 bp ladder; Lane 6, *S. infantis *(patient group); Lanes 7 and 9, *S. ohio *(patient group). For both electrophoresis, run conditions were 120 V, 94 mA, 45 min.

To detect *Salmonella *spp., two separate reactions containing primers complementary to all internal variable regions of the *fliC *and *flj*B genes, which codifies phase I and phase II flagellar antigens (Fig. 2), were used. Both multiplex PCR systems have been previously tested in hundreds of samples from different parts of the world, and were reported to be accurate, fast and reliable alternative to other traditional diagnostic methods used in the clinical laboratory [9,10]. In our study, both stool sample groups showed *S*. *ohio *and *S*. *typhimurium *as the predominant serovars; nevertheless, many other serovars were present in both groups of samples in a considerable proportion (Table 1), which suggest that *Salmonella *diversity is more in comparison to *E. coli*, for which ETEC was considerably predominant (Table 1). Salmonellosis is one of the biggest challenges to public health all over the world. In the year 2000, a study conducted in Mexico showed *S*. *typhimurium *and *S*. *ohio *as the most and less commonly found serotypes of *Salmonella *respectively [39]. This may be related to the way the outbreak was studied, since we focused on most common serotypes in children (age < 12 years) and not in the whole population. This study is intended to investigate some interesting observations that deserve further studying. The results revealed that all stool samples from the patients and the majority of the control group were positive for at least one enteropathogen and that 52% of stool samples had multiple enteropathogens. All of the 300 stool samples tested except 2 had either *E. histolytica/E. dispar *or *G. intestinalis *and when an enterobacteria was present, it was most likely to be a *Salmonella *serotype. In addition, only 12 out of 300 stool samples tested *E. coli *presence was in association with a protozoa in the absence of any type of *Salmonella *spp. (Table 2).

It has been reported that *S*. *typhimurium *virulence is enhanced when exposed to the rumen protozoa [40]; this hypervirulent phenotype is linked to the presence of the *Salmonella *genomic island SGI1 integron [41]. Also, it has been shown that SGI1 is widely spread in other *S. enterica *serovars; and although rumen possesses different protozoa diversity than the parasites studied here, some *Salmonella *isolates from human infections have shown to posses such island [42]. It is tempting to speculate that a similar virulence enhancement process might occur in human digestive trait; possibly through *E. histolytica/E. dispar *or *G. intestinalis*. Whether those *Salmonella *strains studied in this work possesses or expresses such genomic island needs further study.

## Conclusion

The study of enteropathogen associations may lead to a better understanding of the etiology of diarrhoea, and therefore in prescribing more suitable treatments in cases of outbreaks. This study revealed that 52% of the patients were infected by more than one enteropathogen, notably *E. histolitica*/*E. dispar *and *Salmonella ohio*. These results are useful for clinicians in improving the empiric treatment used in such cases.

## Competing interests

The author(s) declare that they have no competing interests.

## Authors' contributions

GLP and EM carried out the molecular studies; OG, JA and EN characterized bacterial isolates, and identified the parasites; SV conceived of the study, and participated in its design and coordination, and drafted the manuscript. All authors read and approved the final manuscript.
